# SOX9, GATA3, and GATA4 Overexpression in Liposarcomas: Insights into the Molecular Biology of Adipocytic Sarcomas

**DOI:** 10.3390/ijms262210981

**Published:** 2025-11-13

**Authors:** Andrei-Ionuț Patrichi, Zsolt Kovács, Ioan Jung, Simona Gurzu

**Affiliations:** 1 Department of Pathology, George Emil Palade University of Medicine, Pharmacy, Science and Technology, 540139 Targu-Mures, Romania; patrichi_andrei@yahoo.ro (A.-I.P.); jungjanos@studium.ro (I.J.); 2 Department of Biochemistry, George Emil Palade University of Medicine, Pharmacy, Science and Technology, 540139 Targu-Mures, Romania; kovacska_zsoltkovacs@yahoo.com; 3 Research Center of Oncopathology and Translational Research (CCOMT), 540139 Targu-Mures, Romania; 4 Romanian Academy of Medical Sciences, 030167 Bucuresti, Romania

**Keywords:** SOX9, GATA3, GATA4, liposarcoma, gene expression

## Abstract

Liposarcomas represent a heterogeneous group of malignant mesenchymal neoplasms, with diverse histological subtypes and molecular alterations. This study aimed to investigate the gene expression profiles of SOX9, GATA3, and GATA4 in liposarcoma subtypes and to assess their associations with clinicopathological parameters. Forty-two formalin-fixed, paraffin-embedded liposarcoma samples were analyzed. Total RNA was extracted, reverse-transcribed, and quantified by qRT-PCR using GAPDH as an endogenous control. Relative quantification (RQ) values were categorized, and statistical analyses included Fisher’s exact test, Kaplan–Meier survival analysis, and Cox proportional hazards modeling. SOX9 expression significantly varied among histological subtypes (*p* = 0.017), with ALT/WDLS cases showing a predominance of high-level expression (RQ > 50 in 12/15 cases), in contrast to myxoid subtypes clustering mainly in the 10–50 RQ range. GATA4 overexpression correlated with smaller tumor size (<100 mm) (*p* = 0.049), being more frequent in 15/20 small tumors compared to 10/22 larger ones. GATA3 and GATA4 demonstrated the strongest inter-gene correlation (r = 0.68, *p* < 0.05), suggesting possible functional interplay. Kaplan–Meier analysis revealed no statistically significant survival differences for individual gene expression, but a high combined GATA3–GATA4 signature was associated with a favorable trend. These findings indicate that SOX9, GATA3, and GATA4 are broadly upregulated in liposarcomas, with subtype- and size-dependent expression patterns. The strong association between GATA3 and GATA4 expression supports their potential synergistic role in tumor biology. Integration of these molecular markers into diagnostic and prognostic workflows may enhance subtype characterization and inform targeted therapeutic strategies. Further studies in larger cohorts are warranted to validate these biomarkers and explore their mechanistic interplay in liposarcoma pathogenesis.

## 1. Introduction

Liposarcoma represents one of the most frequent histological subtypes of soft tissue sarcoma in adults, accounting for approximately 20% of all cases [1]. These tumors typically arise in deep soft tissue compartments, with a predilection for the lower extremities and retroperitoneum—sites that host up to 24% and 45% of limb and retroperitoneal sarcomas, respectively [2,3]. According to the most recent World Health Organization (WHO) Classification of Soft Tissue and Bone Tumours (5th Edition, 2020), liposarcomas are subdivided into five histological subtypes—atypical lipomatous tumor/well-differentiated liposarcoma (ALT/WDLS), dedifferentiated liposarcoma (DDLPS), myxoid liposarcoma (MLS), pleomorphic liposarcoma (PLS), and myxoid pleomorphic liposarcoma (MPLS)—which reflect distinct morphological and molecular features corresponding to different stages of adipocytic differentiation [4,5]. Although the current histopathological classification provides a reliable framework for diagnosing liposarcomas, there remain significant challenges in accurately distinguishing between certain subtypes, especially in cases with limited differentiation or overlapping morphological features [6,7]. For instance, dedifferentiated liposarcomas can mimic other high-grade sarcomas, and pleomorphic forms often lack specific histological hallmarks. Moreover, conventional immunohistochemical markers such as MDM2 and CDK4, while useful in well-differentiated and dedifferentiated subtypes, offer limited utility in the broader molecular landscape of these tumors [8,9]. In this context, molecular profiling based on gene expression analysis has emerged as a promising approach to refine diagnostic accuracy, uncover biologically meaningful subgroups, and identify potential therapeutic targets [10,11].

Transcription factors play a central role in controlling cellular differentiation, proliferation, and lineage commitment, and aberrant expression of these regulators is increasingly recognized as a hallmark of various mesenchymal and epithelial malignancies [12]. Evaluating the expression patterns of specific transcription factors in liposarcomas may therefore provide insights not only into tumor classification but also into the mechanisms that drive tumorigenesis in adipocytic sarcomas [13]. The quantitative assessment of gene expression, particularly via real-time PCR (qRT-PCR), enables precise, reproducible measurement of mRNA levels, and allows detection of subtle molecular alterations that may escape histological detection [14,15]. Relative quantification methods using reference genes further strengthen the comparability across samples and subtypes [16].

Despite progress in molecular diagnostic tools, the current literature offers limited data regarding the expression profiles of transcription factors such as the Sex-determining region Y-box 9 (SOX9), GATA3, and GATA4 in liposarcoma.

SOX9 is a transcription factor belonging to the SOX family of genes, named for their homology to the sex-determining region of the Y chromosome (SRY). It plays a critical role in chondrogenesis, cartilaginous matrix differentiation in developing heart valve structures, sex differentiation, and the maintenance of stem and progenitor cell populations. Similarly, SOX9 and GATA-4 (GATA-binding protein 4) are known as markers involved in the remodeling process of the mesenchymal tissue, during the aging process [17,18]. SOX9 regulates the expression of genes involved in extracellular matrix production, epithelial-to-mesenchymal transition (EMT), and cell cycle progression. Its overexpression has been reported in various malignancies, including hepatocellular carcinoma, glioma, colorectal cancer, and prostate cancer, where it is associated with increased tumor aggressiveness and resistance to therapy [17,18,19,20,21,22,23].

GATA-binding protein 3 (GATA3) is a zinc finger transcription factor essential for the development of various epithelial tissues, particularly the mammary gland and the urothelium. It also serves as a master regulator in the differentiation of T-helper 2 (Th2) lymphocytes. In breast cancer, GATA3 is frequently used as a diagnostic and prognostic marker, and its expression has been associated with favorable outcomes in hormone receptor-positive subtypes. Moreover, GATA3 has also been detected in several other malignancies, including urothelial carcinoma, salivary gland tumors, and subsets of T-cell lymphomas [17,24,25,26,27,28].

GATA4, another member of the GATA family, is primarily expressed in mesoderm-derived tissues such as the heart, gonads, and gastrointestinal tract. It is involved in early embryonic development, regulation of apoptosis, and tissue remodeling. In the context of cancer, GATA4 has been shown to exert context-dependent functions—acting as a tumor suppressor in gastric and colon cancers, while displaying oncogenic behavior in ovarian and pancreatic tumors [20,29,30]. Its dual functional potential highlights the importance of tumor-specific investigations [31].

Although these transcription factors have been mainly investigated in epithelial malignancies, several recent studies have highlighted their biological relevance in soft tissue and mesenchymal tumors. For instance, GATA3 expression was detected in various soft tissue sarcomas (STS) and was correlated with unfavorable clinicopathological parameters and reduced disease-free survival [17]. In tumors, SOX9 was described to be implicated in chordoma and osteosarcoma, where its overexpression promotes invasion and proliferation, supporting a role in endothelial-mesenchymal transition [19]. GATA4, although less studied in sarcomas, participates in mesodermal and myogenic transcriptional networks, regulating cellular plasticity and stress responses, interacting with GATA4 during mesenchymal tissue remodeling [18,20]. Together, these data justify investigating SOX9, GATA3, and GATA4 within the mesenchymal context of liposarcoma, where they may cooperatively contribute to aberrant lineage maintenance and tumor progression. These genes encode nuclear proteins that bind specific DNA sequences to regulate transcriptional programs critical for cell fate determination, proliferation, and lineage-specific differentiation.

Considering these knowledge limitations, the present study aims to evaluate the relative expression levels (RQ) of SOX9, GATA3, and GATA4 in a series of human liposarcoma samples, using a comparative molecular approach based on quantitative real-time PCR. Relative quantification (RQ) is a widely accepted method in molecular oncology that enables accurate and reproducible assessment of gene expression, normalized to reference genes, and allows for direct comparison across samples and biological conditions [15]. This type of analysis is particularly useful in soft tissue sarcomas, where histological overlap and variability in tissue composition can obscure subtle but biologically meaningful transcriptional changes. Through this molecular profiling effort, our study seeks to identify whether consistent overexpression of one or more of these transcription factors can be observed in liposarcomas. Furthermore, we explore potential co-expression patterns or correlations between SOX9, GATA3, and GATA4, which may reflect shared regulatory pathways or converging roles in tumor differentiation, plasticity, or stromal remodeling. Such correlations, if present, could suggest a cooperative function in sustaining a dedifferentiated or progenitor-like cellular state, as previously proposed in other mesenchymal tumors [32]. Clinically, elucidating the transcriptional landscape of liposarcomas may aid in developing molecular classifiers capable of supplementing conventional histopathological diagnosis. From a therapeutic perspective, aberrant activation of lineage-specific transcription factors could uncover vulnerabilities that may be exploited pharmacologically, either through direct targeting or by interfering with downstream signaling networks. Although these genes are not currently therapeutic targets in liposarcoma, prior studies in breast, liver, and gastrointestinal cancers have suggested that modulating transcription factor activity may influence tumor progression, treatment response, and recurrence risk [33,34,35]. To our knowledge, this is the first study that systematically quantify and compare SOX9, GATA3, and GATA4 expression in liposarcoma specimens using qRT-PCR-based relative quantification. By establishing a molecular baseline for these factors in adipocytic sarcomas, our findings are expected to enrich the current understanding of liposarcoma biology and encourage a more integrated, gene-informed approach to diagnosis, classification, and potentially, targeted therapy.

## 2. Results

### 2.1. Expression Patterns of SOX9, GATA3, and GATA4 in Liposarcoma Cases

The relative expression values (RQ) of SOX9, GATA3, and GATA4 were evaluated in 42 liposarcoma cases. Descriptive statistics revealed a wide range of RQ values for all three genes, with SOX9 showing a mean of 47.38 (SD 27.54), GATA3 a mean of 47.26 (SD 28.71), and GATA4 the highest mean of 53.50 (SD 29.54) (Table 1).

**Figure 1:**
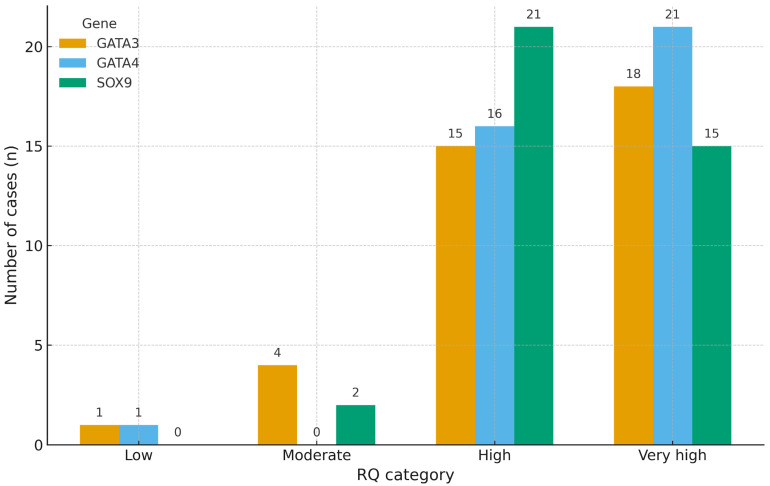
Distribution of RQ-based gene expression levels in liposarcoma samples. The stacked bar chart illustrates the relative frequency of expression categories for SOX9, GATA3, and GATA4. The number of cases (n) corresponding to each category is indicated within the respective bar segments. Expression thresholds were defined as low (RQ < 1), moderate (RQ 1–10), high (RQ 10–50), and very high (RQ > 50).

Among the 42 analyzed liposarcoma cases, a predominant proportion exhibited elevated expression levels for all three genes. For SOX9, 26 cases (61.9%) were classified as having either high (n = 15) or very high (n = 11) expression. An identical pattern was observed for GATA3, with 14 cases in the high and 12 in the very high category, totaling 26 cases (61.9%). GATA4 showed a slightly broader distribution, with 13 cases (31.0%) in the moderate category, 15 (35.7%) in the high, and 11 (26.2%) in the very high range, suggesting a more heterogeneous overexpression profile. In contrast, low expression levels were observed in only 3 cases for SOX9, 2 for GATA3, and 2 for GATA4. Overall, these results support a consistent overexpression trend across all three genes, with inter-gene variability in the relative moderate versus very high expression (Figure 1).

Boxplot analysis confirmed substantial interindividual variability in the expression levels of SOX9, GATA3, and GATA4 across the 42 analyzed liposarcoma cases. The median RQ values were comparable among the three genes: SOX9—42.11, GATA3—42.07, and GATA4—53.34. The interquartile range (IQR) was widest for GATA4, suggesting greater heterogeneity in its overexpression profile (IQR = 45.57), followed by GATA3 (IQR = 38.81) and SOX9 (IQR = 36.74). All three genes reached their highest relative expression values close to 100—specifically, 97.39 for SOX9, 98.14 for GATA3, and 99.81 for GATA4—indicating that some samples exhibited expression levels approximately 100-fold higher than the calibrator (RQ = 1). These values represent the upper range of relative quantification within this cohort rather than a technical limit of detection, as all qPCR reactions remained within the linear amplification range. The presence of outliers in each distribution further emphasized the marked heterogeneity in gene expression across the tumor samples.

### 2.2. Gene Expression Interaction Analysis of SOX9, GATA3, and GATA4

Correlation analysis between gene expression levels revealed significant positive associations between the expression levels of the three genes (Table 2). The strongest correlation was observed between GATA3 and GATA4 (r = 0.571, *p* < 0.001), suggesting a potential co-regulatory pattern. Another correlation was also noted between SOX9 and GATA3 (r = 0.477, *p* = 0.002), while the association between SOX9 and GATA4 was weaker but still statistically significant (r = 0.314, *p* = 0.045). These findings support the presence of partially overlapping expression dynamics among the studied genes in liposarcoma. While these associations do not confirm direct co-regulation, they suggest the presence of partially convergent expression trends across certain gene pairs in the analyzed liposarcoma cases.

To further evaluate the relationships between gene expression profiles, scatterplots were generated for each gene pair: SOX9 vs. GATA3, SOX9 vs. GATA4, and GATA3 vs. GATA4. In the SOX9–GATA3 plot, data points are widely scattered, with a slight upward trend that aligns with the moderate correlation coefficient (r = 0.477). The SOX9–GATA4 panel exhibits an even more diffuse pattern, with considerable dispersion at both low and high RQ values, consistent with the weaker correlation observed (r = 0.314). The GATA3–GATA4 comparison displays the most noticeable concentration of points along a diagonal trajectory, reflecting the strongest correlation in the dataset (r = 0.571), although substantial spread is still present. The graphical distribution of data points does not suggest a strong linear clustering in any of the comparisons. However, the observed patterns are generally in line with the statistical significance reported in the Spearman analysis, indicating that while expression levels of certain gene pairs may trend together, these associations remain variable. Overall, the scatterplots highlight heterogeneous expression dynamics without pointing to sharply defined co-expression subsets (Table 2).

### 2.3. Correlation of SOX9, GATA3, and GATA4 Expression with Clinicopathological Features

Statistical analysis using Fisher’s exact test identified two clinicopathological parameters that demonstrated significant associations with gene expression levels. SOX9 expression varied significantly among histological subtypes (*p* = 0.017), with ALT/WDLS cases exhibiting a predominance of high-level expression (RQ > 50 in 12 out of 19 cases), in contrast to myxoid subtypes, which predominantly clustered within the 10–50 RQ range (RQ 10–50 in 8 out of 9 cases). Additionally, GATA4 expression was significantly correlated with tumor size (*p* = 0.049), being more frequently overexpressed in tumors measuring less than 100 mm in maximum diameter (15 out of 20 cases with RQ > 50), compared to those exceeding 100 mm (10 out of 22 cases). These findings suggest subtype- and size-related heterogeneity in gene expression patterns within the liposarcoma cohort.

In contrast, when examining the broader set of clinicopathological variables, no statistically significant associations were identified between the expression levels of SOX9, GATA3, or GATA4 and features such as gender, patient age, tumor localization, histologic grade, tumor stage, inflammation, fibrosis, mitotic index, resection margin status, or recurrence (all *p* > 0.05). Specifically, gene expression did not vary significantly with gender (SOX9: *p* = 0.25; GATA3: *p* = 0.69; GATA4: *p* = 0.45), age group (<60 vs. ≥60 years; *p* range = 0.29–0.87), or tumor site (trunk vs. extremities; *p* range = 0.38–0.90). No significant correlations were observed with histologic grade (*p* = 0.16–0.88), tumor stage (*p* = 0.60–0.89), mitotic activity (*p* = 0.31–0.84), or resection margin status (*p* = 0.61–0.93). Similarly, no statistically significant differences in expression levels were observed in relation to recurrence status for any of the analyzed genes, with *p*-values ranging from 0.26 to 0.47.

Although several non-significant patterns were observed (e.g., apparent tendencies between gene expression and certain clinicopathological parameters), these findings should be interpreted cautiously as exploratory trends rather than established associations. Only correlations with *p* < 0.05 were considered statistically significant, while all other observations are reported here for descriptive completeness and to guide future hypothesis-driven studies in larger cohorts.

The full clinicopathological association table is provided in Supplementary Table S1. Only statistically significant findings are reported in the main text.

### 2.4. Survival Analysis

Kaplan–Meier survival analysis was conducted to evaluate the prognostic relevance of gene expression levels for SOX9, GATA3, and GATA4, as well as a combined expression categories. Although none of the comparisons reached statistical significance based on the log-rank test, several consistent visual trends were observed across expression categories.

For GATA4, the group with low expression (RQ < 1) demonstrated the most favorable survival curve, remaining stable throughout the follow-up period. In contrast, both the 10–50 and >50 RQ groups exhibited earlier declines in survival probability, though the curves largely overlapped (*p* range = 0.2853–0.4205). Similarly, in GATA3, cases with very high expression (RQ > 50) showed the steepest decline in survival, while intermediate and low expression categories appeared to be associated with better outcomes, despite a lack of statistical significance (*p* range = 0.2371–0.5100). In the case of SOX9, survival was most prolonged in the 1–10 RQ group, while both extremes—<1 and >50—showed comparatively reduced survival probabilities, suggesting a potential non-linear or bimodal effect, though again not reaching significance (*p* = 0.1998–0.6093). When integrating all three markers into a combined gene expression score, patients in the 50–100 range displayed the most favorable survival profile, whereas those in the >100 category experienced steeper declines. Although differences did not achieve statistical significance (*p* range = 0.1573–0.5421), this pattern may reflect a threshold beyond which concurrent overexpression of all three genes could relate to poorer prognosis. While these findings do not support a statistically significant prognostic value for individual or combined gene categories in this cohort, the recurrent visual separation of survival curves suggests biologically relevant trends that merit further investigation in larger datasets.

To assess the prognostic relevance of SOX9, GATA3, and GATA4 gene expression levels on overall survival in liposarcoma patients, we performed univariate Cox proportional hazards regression using continuous RQ values as covariates.

None of the three genes exhibited a statistically significant association with overall survival. Specifically, SOX9 expression showed no significant impact on survival outcomes (coefficient = 0.0036, *p* = 0.590), and the 95% confidence interval for the hazard ratio included the null value, suggesting no meaningful effect. Similarly, GATA3 expression demonstrated a non-significant trend toward increased risk (coefficient = 0.0049, *p* = 0.486), while GATA4 expression appeared inversely related to hazard but also failed to reach statistical significance (coefficient = –0.0047, *p* = 0.559).

These data indicate that individual gene expression levels of SOX9, GATA3, and GATA4 do not independently predict survival outcomes in this patient cohort, at least when modeled as continuous variables in a univariate Cox framework.

## 3. Discussion

In this study, we conducted a comprehensive molecular analysis of SOX9, GATA3, and GATA4 gene expression in a cohort of 42 liposarcoma cases using quantitative real-time PCR. Our results revealed a consistent trend of relative upregulation across all three genes within the liposarcoma cohort, with RQ values exceeding 10 in the majority of cases and reaching the upper range of the internal quantification scale (RQ ≈ 100). These findings indicate elevated transcriptional activity relative to the internal reference sample rather than confirmed overexpression versus normal adipose tissue, which remains to be validated in future comparative studies. Such overexpression patterns may reflect an active role of these genes in the molecular landscape of liposarcomas, regardless of the histological subtype. Notably, although SOX9, GATA3, and GATA4 have been previously implicated in various malignancies, this is the first qRT-PCR-based study to systematically quantify their expression in human liposarcoma tissue. The widespread overexpression observed suggests that these transcription factors are likely to participate in shared regulatory networks contributing to tumor development or maintenance. The biological relevance of these findings is further enhanced by the observation that, despite histological and clinical heterogeneity within the cohort, the transcriptional activation of these genes appeared to be a shared feature. This raises the possibility that SOX9, GATA3, and GATA4 are not merely subtype-specific markers but may instead reflect a broader oncogenic pathway active across diverse liposarcoma variants. In agreement with previous reports, our data expand on earlier evidence of SOX9, GATA3, and GATA4 dysregulation in soft tissue sarcomas. Haraguchi et al. demonstrated GATA3 immunopositivity in approximately 20% of STS cases, correlating with higher grade and worse survival, thereby suggesting a broader oncogenic function of this factor beyond epithelial differentiation [17]. Likewise, SOX9 expression was shown to drive mesenchymal proliferation and extracellular matrix remodeling in chordoma and osteosarcoma models, while GATA4 has recently been linked to myogenic reprogramming and mesodermal lineage persistence [19,20].

These findings collectively support our hypothesis that the coordinated transcriptional activation of these three genes represents a shared regulatory axis active within adipocytic sarcomas.

### 3.1. Heterogeneity of GATA4 Expression

Among the three genes analyzed, GATA4 exhibited the most heterogeneous expression pattern across the liposarcoma cohort. Although it showed the highest mean RQ value (53.50), it also presented the widest interquartile range (IQR = 45.57) and the most even distribution across all expression categories. While SOX9 and GATA3 were more frequently confined to the high or very high expression levels, GATA4 was more broadly distributed, suggesting substantial interindividual variability in its expression. Boxplot analysis confirmed this finding, showing a broad spread of values and multiple outliers. This variability may indicate differential transcriptional regulation of the GATA4 gene, potentially influenced by tumor subtype, epigenetic alterations, or tumor microenvironment dynamics. Unlike SOX9 or GATA3, GATA4 did not show a strong expression bias toward any specific liposarcoma subtype or clinicopathological feature.

This heterogeneity is also consistent with the context-dependent biological roles described for GATA4 in other cancers. In gastric and colon tumors, GATA4 has been reported to exhibit tumor-suppressive functions, whereas in ovarian and pancreatic cancers it appears to act in an oncogenic capacity [29,30]. Such duality may also apply in liposarcoma, where its expression could reflect either a differentiation-associated role or an active involvement in tumor progression.

Furthermore, GATA4 expression showed significant correlation with both SOX9 and GATA3, but its distinct transcriptional regulatory associations, including predicted modulation by MYC and NANOG, suggest that GATA4 may also function independently within broader regulatory circuits. Overall, the heterogeneous expression profile of GATA4 supports its potential involvement in diverse transcriptional programs relevant to liposarcoma pathogenesis.

### 3.2. Complementary Roles and Reciprocal Potentiation of GATA3 and GATA4

One of the most notable observations in this study is the strong positive correlation between GATA3 and GATA4 mRNA expression (Spearman r = 0.571, *p* < 0.001), the highest among all pairwise gene comparisons. This finding suggests a coordinated transcriptional pattern for these two GATA family members across liposarcoma samples. Given their structural homology and related DNA-binding motifs, overlapping expression is biologically plausible and may reflect convergent functional roles.

GATA3 is classically involved in epithelial differentiation and immune regulation, yet recent studies have highlighted context-dependent roles in mesenchymal tumors [24,25]. GATA4, although predominantly associated with mesodermal development and regenerative programs, has also shown heterogeneous behavior in cancer biology, acting as either a tumor suppressor or oncogene depending on cellular context [29,30]. Their parallel upregulation in our dataset may indicate shared upstream regulation or convergence on common biological pathways relevant to liposarcoma behavior, including differentiation control, stress-response signaling, and cellular plasticity.

Supporting this possibility, in silico miRNA analysis identified several candidate microRNAs predicted to co-regulate both GATA3 and GATA4 (Table 3). Among these, miR-34a-5p, miR-15a-5p, and miR-200a-3p are recognized regulators of epithelial–mesenchymal transition, apoptosis, and stemness reprogramming in solid tumors [36]. While exploratory, this shared miRNA regulatory signature suggests that GATA3 and GATA4 may participate in a coordinated transcriptional module in liposarcoma.

Collectively, these observations raise the hypothesis that GATA3 and GATA4 act in parallel or complementary fashion to support phenotypic adaptability or dedifferentiation programs in liposarcoma. Whether this reflects hierarchical signaling or a cooperative regulatory axis remains to be established in functional studies.

### 3.3. Transcriptional Regulatory Network of SOX9, GATA3, and GATA4

To gain additional insight into the upstream transcriptional regulation of the studied genes, we used the Gene–TF interaction module within the miRNet platform. The resulting network highlighted distinct and partially overlapping transcription factor (TF) interactions for SOX9 and GATA4, while no validated TF associations were detected for GATA3 under the current database parameters.

SOX9 was found to interact with a panel of eight predicted regulators, including SP1, SRY, SF1, ZBTB16, and RELA. These TFs are known to participate in chromatin remodeling, sex differentiation, and inflammatory signaling. In particular, RELA, a core component of the NF-κB pathway, and SP1, a pleiotropic transcription factor with oncogenic potential, are widely implicated in tumor progression, proliferation, and immune evasion [31]. Their predicted involvement in SOX9 regulation supports its potential contribution to inflammation-driven or stress-responsive signaling cascades in liposarcoma.

GATA4 exhibited interactions with seven regulators, most notably MYC, NANOG, HEY1, HEY2, and NKX2-5—transcription factors associated with pluripotency, stem cell maintenance, and mesodermal lineage specification. The predicted influence of MYC and NANOG is particularly relevant, as these TFs are central players in oncogenic reprogramming and have been shown to mediate stem-like phenotypes in various sarcomas and solid tumors [33].

In contrast, GATA3 did not yield any validated TF associations in this analysis. This may reflect either an actual independence from classical upstream transcriptional control, or a limitation of the current dataset. GATA3 expression may be governed by epigenetic, enhancer-mediated, or post-transcriptional mechanisms not yet fully captured in public interaction databases.

Taken together, these results indicate that SOX9 and GATA4 are embedded within broader transcriptional regulatory networks that are tightly linked to cellular identity, inflammatory stress responses, and dedifferentiation. The absence of TF associations for GATA3, despite its strong expression and correlation with GATA4, suggests a potentially distinct regulatory trajectory that may be decoupled from conventional transcriptional control.

In addition to their oncogenic implications, SOX9, GATA3, and GATA4 play essential roles in mesenchymal stem cell (MSC) differentiation and adipogenic regulation.

SOX9 promotes chondrogenic and osteogenic differentiation while suppressing adipocyte formation, maintaining progenitor multipotency [37]. GATA3 acts as a transcriptional inhibitor of adipogenesis by repressing PPARγ-dependent pathways and sustaining stem cell identity, while also interacting with chromatin remodelers to influence adipose tissue remodeling [38,39]. Conversely, GATA4 operates as an early transcriptional activator of adipogenic genes, driving adipocyte lineage commitment and lipid accumulation [40].

Collectively, these findings underscore that the altered expression of SOX9, GATA3, and GATA4 observed in liposarcoma may reflect dysregulated MSC differentiation mechanisms and impaired adipogenic transcriptional control.

It is also important to note that other transcription factors, such as GATA2, are known regulators of adipogenesis. GATA2 suppresses adipogenic differentiation by directly inhibiting PPARγ transcriptional activity and maintaining the preadipocyte state [41]. Our study specifically focused on GATA3 and GATA4, which share structural similarity with GATA2 but exhibit partially distinct regulatory functions in mesenchymal and adipogenic contexts. In contrast to GATA2, which primarily acts as an upstream repressor of adipogenesis, GATA3 and GATA4 are dynamically modulated during differentiation and have been implicated in the transcriptional plasticity of mesenchymal tumors. Therefore, these genes were selected to investigate whether their expression reflects altered mesenchymal lineage control in liposarcomas rather than canonical adipogenic suppression alone.

Exploratory transcription factor prediction results are provided in Supplementary Figure S1.

### 3.4. High Expression in Low-Grade Liposarcoma Subtypes: ALT/WDLS

An important and somewhat counterintuitive observation in our dataset was the frequent overexpression of SOX9 and GATA4 in ALT/WDLS cases, which are classically regarded as low-grade or well-differentiated liposarcoma subtypes. Specifically, 12 out of 19 ALT/WDLS tumors showed very high expression of SOX9 (RQ > 50), while GATA4 also exhibited elevated levels in the majority of these cases. These findings suggest that the activation of these genes is not limited to high-grade or dedifferentiated variants, but rather may represent early molecular events in liposarcomagenesis.

This hypothesis is supported by recent literature. A study by Merry et al. emphasized that transcriptional activation of stemness- and development-related pathways is not confined to dedifferentiated tumors but can also be detected in well-differentiated liposarcomas, possibly reflecting preneoplastic alterations or lineage-based programs maintained from early tumor stages [29]. Similarly, Yang et al. reported that molecular aberrations associated with adipocytic differentiation—including dysregulated transcription factors—may be detectable even in low-grade tumors and could contribute to their potential for progression or recurrence [1].

In our data, this trend is most pronounced for SOX9, a gene known for its role in maintaining progenitor states and regulating extracellular matrix components. Its persistent expression in ALT/WDLS may point to an ongoing, non-physiological activation of differentiation-inhibitory pathways, which could prevent adipocytic maturation while promoting tumor cell survival.

For GATA4, its association with smaller tumor size and overexpression in tumors < 100 mm suggests that it may act as an early-response gene, possibly involved in modulating growth signals or angiogenic responses before morphological dedifferentiation becomes apparent. Such a pattern is consistent with the proposed role of GATA4 in tissue remodeling and regenerative stress seen in other solid tumors [27].

Thus, while ALT/WDLS tumors are generally associated with favorable prognosis, our data indicate that transcriptional activation of specific gene programs may precede histological progression and could be used to stratify cases with higher molecular aggressiveness even within this histologically “benign” group. These findings underscore the importance of integrating molecular profiling into the risk assessment and classification of liposarcomas.

### 3.5. Survival Trends: Biological Signals Beyond Statistical Significance

Although Kaplan–Meier analysis suggested subtle separation of survival curves across gene expression categories, none of these trends reached statistical significance and they should be interpreted as exploratory observations. However, several reproducible and biologically suggestive trends emerged from the data. For GATA3, patients with very high gene expression (RQ > 50) showed the steepest decline in survival probability, while cases with moderate or low expression had more favorable trajectories. A similar pattern was observed in GATA4, where the highest expression group experienced early survival drops compared to the <1 RQ group, which showed the most stable outcomes. These trends may reflect a dose-dependent oncogenic effect, whereby excessive activation of GATA-driven pathways contributes to aggressive tumor behavior or resistance to therapy.

Interestingly, SOX9 exhibited a non-linear relationship with survival. The best outcomes were observed in the moderate expression group (RQ 1–10), whereas both low (<1) and very high (>50) expression groups showed comparatively worse survival. This bimodal pattern may reflect context-specific roles of SOX9: at moderate levels, it could support cellular homeostasis or differentiation, while overexpression may promote tumorigenesis via extracellular matrix remodeling or anti-apoptotic mechanisms [17,19].

Moreover, when analyzing the combined gene expression signature (based on total RQ values across SOX9, GATA3, and GATA4), patients in the intermediate range (50–100) exhibited the most favorable survival profile. In contrast, those with the highest combined expression scores (>100) experienced the steepest declines. Although these differences did not reach statistical significance (*p* > 0.15), the recurrent separation of survival curves suggests an underlying biological gradient that may become apparent in larger, adequately powered cohorts.

Similar observations have been reported in other sarcoma subtypes. For instance, Bushweller et al. and Shiah et al. discussed how transcriptional amplifications of key developmental regulators can be associated with tumor aggressiveness, even when not captured by classic grading systems [30,31]. In this context, high-level gene activation may not be an epiphenomenon but rather a driver of poor prognosis, warranting further exploration in future studies with extended follow-up and molecular stratification. Taken together, although our survival analysis did not yield statistically robust prognostic markers, the consistency of expression-associated trends across individual and combined gene models suggests that transcriptional profiling may provide added biological insight that complements traditional risk factors.

## 4. Materials and Methods

### 4.1. Case Selection and Inclusion Criteria

A total of 42 liposarcoma cases diagnosed between 2016 and 2023 were selected from the archives of the Department of Pathology, Clinical County Emergency Hospital, Târgu Mureș, Romania. Inclusion criteria required a confirmed histopathological diagnosis of liposarcoma (any histological subtype), the availability of sufficient tumor material in formalin-fixed paraffin-embedded (FFPE) tissue blocks, and the absence of neoadjuvant therapy prior to surgical excision. Exclusion criteria included cases with extensive tissue necrosis, degraded nucleic acids incompatible with RNA extraction, or samples with insufficient tumor cellularity for reliable molecular analysis. All cases were reviewed by a senior pathologist (SG) to confirm diagnostic accuracy and tissue adequacy prior to molecular processing. The study was reviewed and approved by the Ethics Committee of the Clinical County Emergency Hospital—Târgu Mureș, Romania (Decision no 14967/16 June 2021).

The cohort included all liposarcoma histological subtypes: atypical lipomatous tumor (ALT)/well-differentiated liposarcoma (WDLS), dedifferentiated liposarcoma (DLS), myxoid liposarcoma (MLS), pleomorphic liposarcoma (PLS), and myxoid pleomorphic liposarcoma (MPLS), classified according to the 2020 WHO criteria [4,5].

All tumor specimens represented primary liposarcomas obtained at the time of initial surgical resection. None of the patients had received neoadjuvant chemotherapy or radiotherapy prior to surgery. Adjuvant systemic therapy, when administered, followed standard soft tissue sarcoma protocols (doxorubicin ± ifosfamide regimens); however, no molecular analyses were performed on post-treatment specimens.

### 4.2. RNA Extraction and cDNA Synthesis

Total RNA was extracted from deparaffinized FFPE sections using a commercially available kit (e.g., RNeasy FFPE Kit, Qiagen, Hilden, Germany) in accordance with the manufacturer’s instructions. The concentration and purity of the extracted RNA were assessed using a NanoDrop spectrophotometer (Thermo Fisher Scientific, Waltham, MA, USA), and optical density (OD) was evaluated where possible.

Complementary DNA (cDNA) was synthesized from 1 μg of total RNA using the High-Capacity cDNA Reverse Transcription Kit (Applied Biosystems, Waltham, MA, USA) in a final volume of 20 μL, with random hexamer primers, following the manufacturer’s protocol.

### 4.3. Quantitative Real-Time PCR (qRT-PCR)

Gene expression levels of SOX9, GATA3, and GATA4 were assessed using quantitative real-time PCR (qRT-PCR) performed with SYBR Green chemistry on a QuantStudio 3 Real-Time PCR System. All reactions were run in triplicate for each sample to ensure reproducibility.

The housekeeping gene—GAPDH—was used as an endogenous reference for normalization, similar to the methods used for our previous studies [18]. Primer sequences for all genes were sourced from commercially validated assays or relevant literature. Amplification specificity was confirmed by melting curve analysis.

### 4.4. Data Analysis and Statistical Evaluation

Data were initially processed and curated using Microsoft Excel (Office 365). Statistical analyses were performed using GraphPad Prism v9.0 (GraphPad Software, San Diego, CA, USA) and Python (v3.10) within Google Colab. Descriptive statistics included means, standard deviations, and frequency distributions.

Relative expression levels were calculated using the comparative ΔΔCt method, previously described by Livak and Schmittgen and validated by our team [18,42] with all values normalized to GAPDH and expressed as relative quantification (RQ). Fold changes were computed relative to a calibrator sample selected from within the cohort. Regarding the distribution of gene expression levels, each gene’s relative quantification (RQ) values were stratified into four interpretive categories to enhance analytical clarity: low (RQ < 1), moderate (RQ 1–10), high (RQ 10–50), and very high (RQ > 50) expression.

Non-parametric association tests (Chi-square, Fisher’s exact, Mann–Whitney U) were used to assess correlations between gene expression levels (RQ) and clinicopathological parameters. A *p*-value < 0.05 was considered statistically significant. Kaplan–Meier survival analysis was conducted using the Lifelines Python package, with the log-rank test applied for significance. Multivariate survival analysis was performed using Cox proportional hazards regression models to evaluate the effect of individual genes and a combined gene expression signature (termed Signature_High) on overall survival, adjusting for histologic grade and tumor size. Figures were generated using Matplotlib (version 3.10.0) and Seaborn libraries (https://seaborn.pydata.org/ accessed on 20 of August 2025).

### 4.5. miRNA–Gene Interaction Network Analysis

To explore potential regulatory interactions affecting gene expression, the genes SOX9, GATA3, and GATA4 were submitted to the miRNet 2.0 platform [33,34]. First, a miRNA–gene interaction network was generated to identify microRNAs with predicted or validated interactions with the selected genes. Second, the Gene–TF interaction module was applied to construct a transcription factor (TF) network, highlighting potential upstream regulators of SOX9, GATA3, and GATA4. Both networks were visualized through the platform’s integrated graphical tools.

## 5. Conclusions

This study provides qRT-PCR-based evaluation of SOX9, GATA3, and GATA4 gene expression in liposarcomas, revealing consistent overexpression patterns across multiple histological subtypes. Among the key findings, GATA4 emerged as the most heterogeneous gene, suggesting a context-dependent regulatory role, while GATA3 and GATA4 showed the strongest expression correlation, potentially indicating cooperative transcriptional activity. Notably, high expression levels of SOX9 and GATA4 were frequently detected in ALT/WDLS tumors, suggesting that these genes may be activated early in tumorigenesis, even in histologically well-differentiated cases. Although survival analysis did not yield statistically significant associations, consistent biological trends pointed to a dose-dependent relationship between overexpression and poorer outcomes, particularly for GATA3 and GATA4. In addition to expression patterns, our miRNA and transcription factor network analyses uncovered shared regulatory pathways, with GATA4 and SOX9 embedded in transcriptional circuits involving RELA, MYC, and NANOG and jointly targeted by miRNAs implicated in stemness and EMT. These data highlight the potential biological and clinical relevance of these genes in liposarcoma pathogenesis.

Altogether, the findings support the value of integrating gene expression profiling into liposarcoma characterization, with potential implications for molecular classification, prognostic stratification, and future therapeutic exploration. Further validation in larger and functionally annotated cohorts is warranted to fully define the diagnostic and biological utility of SOX9, GATA3, and GATA4 in adipocytic sarcomas.

## Figures and Tables

**Table 1:** Descriptive statistics of relative gene expression (RQ values) for SOX9, GATA3, and GATA4 across 42 liposarcoma cases. The table summarizes the mean, median, standard deviation, and range of RQ values, highlighting the overexpression pattern and interindividual variability observed in the cohort.

Gene	Mean RQ	Median RQ	Std. Deviation	Minimum	Maximum
*SOX9*	47.38	42.11	27.54	1.04	97.39
*GATA3*	47.26	42.07	28.71	1.01	98.14
*GATA4*	53.50	53.34	29.54	1.03	99.81

**Table 2:** Spearman correlation analysis to assess co-expression between SOX9, GATA3, and GATA4.

	SOX9	GATA3	GATA4
SOX9	1	0.477 (*p* = 0.002)	0.314 (*p* = 0.045)
GATA3	0.477 (*p* = 0.002)	1	0.571 (*p* < 0.001)
GATA4	0.314 (*p* = 0.045)	0.571 (*p* < 0.001)	1

**Table 3:** Predicted microRNAs potentially regulating SOX9, GATA3 and GATA4 expression. Bioinformatic analysis identified 12 miRNAs with putative binding affinity to the three transcription factors, several of which have been previously implicated in tumor cell proliferation, apoptosis, epithelial–mesenchymal transition (EMT), and metastatic behavior. These predictions are exploratory and provide hypothesis-generating insights into post-transcriptional regulatory mechanisms relevant to liposarcoma biology.

miRNA	Predicted Target(s)	Reported Biological Function
hsa-miR-423-5p	SOX9/GATA3/GATA4	linked to proliferation and EMT
hsa-miR-19a-3p	SOX9/GATA3	oncogenic, PI3K/AKT signaling
hsa-miR-19b-3p	SOX9/GATA3	oncogenic in soft tissue tumors
hsa-miR-26b-5p	SOX9/GATA4	tumor suppressor, inhibits invasion
hsa-miR-1-3p	GATA4	muscle differentiation, tumor suppression
hsa-miR-30a-5p	SOX9	EMT suppression, apoptosis promotion
hsa-miR-30d-5p	SOX9/GATA3	oncogenic in sarcoma
hsa-miR-15a-5p	GATA4/GATA3	cell cycle arrest, tumor suppressor
hsa-miR-200a-3p	SOX9	inhibits EMT and metastasis
hsa-miR-15b-5p	GATA3	cell cycle regulation
hsa-miR-195-5p	SOX9	induces apoptosis, tumor suppressor
hsa-miR-34a-5p	SOX9/GATA4	p53-regulated tumor suppressor

## Data Availability

The original contributions presented in this study are included in the article/Supplementary Material. Further inquiries can be directed to the corresponding author. The data used and reported in the Discussion section was generated using the online free-access miRNet 2.0 platform: https://www.mirnet.ca/ (accessed on 2 August 2025).

## Supplementary Materials

The following supporting information can be downloaded at: https://www.mdpi.com/article/10.3390/ijms262210981/s1.

## Abbreviations

The following abbreviations are used in this manuscript:

ALT	Atypical Lipomatous Tumor
DDLPS	Dedifferentiated Liposarcoma
EMT	Epithelial–Mesenchymal Transition
FFPE	Formalin-Fixed Paraffin-Embedded
GAPDH	Glyceraldehyde-3-Phosphate Dehydrogenase
GATA3	GATA-binding protein 3
GATA4	GATA-binding protein 4
GPS2	G Protein Pathway Suppressor 2
HEY1	Hairy/Enhancer-of-split related with YRPW motif 1
HEY2	Hairy/Enhancer-of-split related with YRPW motif 2
IQR	Interquartile Range
MDM2	Mouse Double Minute 2 Homolog
MPLS	Myxoid Pleomorphic Liposarcoma
MYC	MYC Proto-Oncogene
NANOG	NANOG Homeobox
NF-κB	Nuclear Factor Kappa-light-chain-enhancer of Activated B cells
NKX2-5	NK2 Homeobox 5
PCR	Polymerase Chain Reaction
PLS	Pleomorphic Liposarcoma
qRT-PCR	Quantitative Real-Time Polymerase Chain Reaction
RQ	Relative Quantification
RELA	RELA Proto-Oncogene, NF-κB Subunit
SF1	Steroidogenic Factor 1
SOX9	SRY-Box Transcription Factor 9
SP1	Specificity Protein 1
SRY	Sex-Determining Region Y
TF	Transcription Factor
WDLS	Well-Differentiated Liposarcoma
ZBTB16	Zinc Finger and BTB Domain Containing 16
