# Supplementary material for: SOX9, GATA3, and GATA4 Overexpression in Liposarcomas: Insights into the Molecular Biology of Adipocytic Sarcomas
Source: Int J Mol Sci. 2025 Nov 13;26(22):10981. doi: 10.3390/ijms262210981 (PMC12652793; doi:10.3390/ijms262210981)
Supplement: Supplementary file 1 [file ijms-26-10981-s001.zip › ijms-3919554-supplementary.pdf]

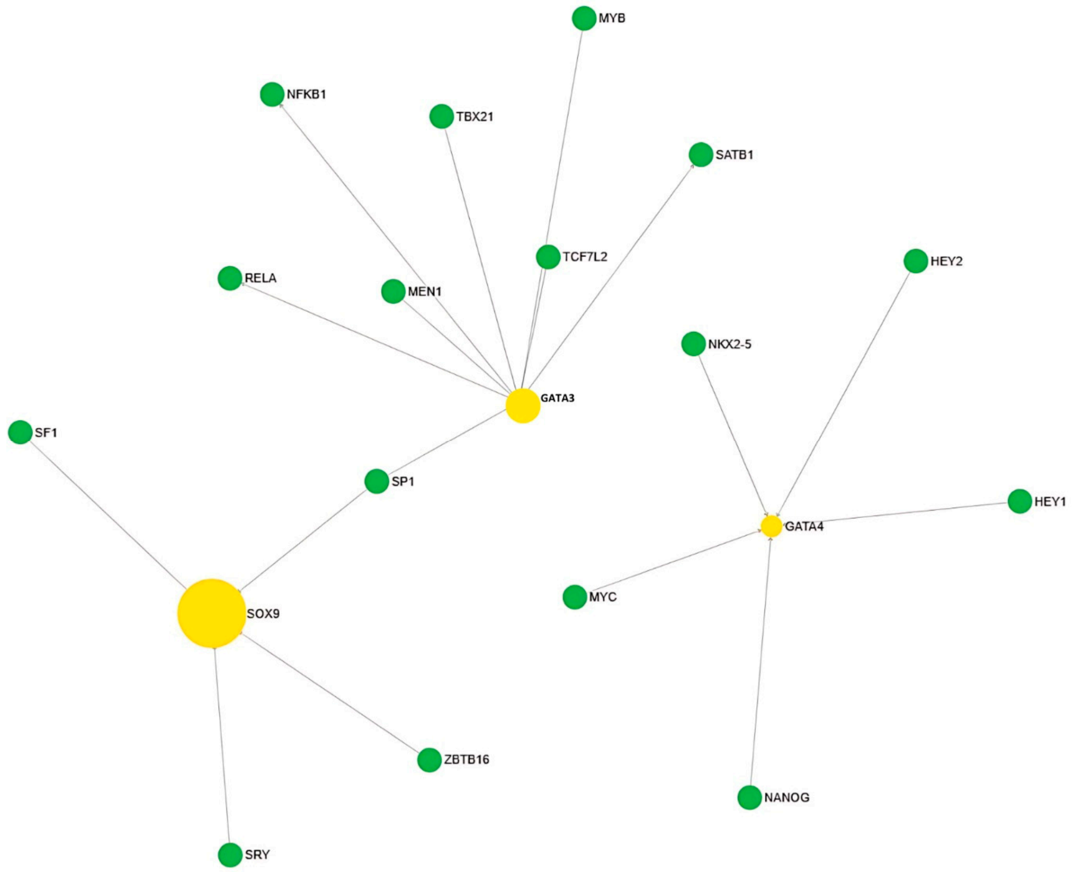

Supplementary Figure S1. Predicted transcription factor interactions for SOX9, GATA3 and GATA4 generated via miRNet. This exploratory visualization is provided to support the discussion and illustrate predicted upstream transcriptional regulators.

Supplementary Table S1. Distribution of SOX9, GATA3, and GATA4 expression across clinicopathological parameters.

Variable		SOX9 (RQ)					GATA3 (RQ)					GATA4 (RQ)				
		<1	1-10	10-50	>50	P value	<1	1-10	10-50	>50	P value	<1	1-10	10-50	>50	P value
Gender	Male	0	1	9	13	0.25	1	3	8	11	0.69	0	0	8	15	0.45
	Female	0	1	12	6		0	1	7	11		1	0	8	10	
Age	<60 years	0	2	8	9	0.29	0	1	9	9	0.49	0	0	7	12	0.87
	≥60 years	0	0	13	10		1	3	7	12		1	0	9	13	
Tumor localization	Trunk	0	2	12	12	0.90	0	3	11	13	0.45	1	0	9	17	0.38
	Extremities	0	0	8	7		1	1	4	9		0	0	7	8	
Maximum tumor diameter	<100 mm	0	2	9	9	0.58	0	1	6	13	0.42	1	0	4	15	**0.049**
	>100 mm	0	0	12	10		1	3	9	9		0	0	12	10	
Histopathological subtype	ALT/WDLS	0	0	7	12	**0.017**	1	2	6	10	0.47	1	0	6	12	0.31
	MYXOID LPS (MLS)	0	0	8	1		0	0	3	6		0	0	3	6	
	PLEOMORPHIC LPS (PLS)	0	1	1	0		0	1	1	0		0	0	0	2	
	DDLPS	0	1	4	6		0	1	5	5		0	0	7	4	
	MYXOID PLEOMORPHIC LS (MPLS)	0	0	1	0		0	0	0	1		0	0	0	1	
Histologic grade	Grade 1	0	0	8	11	0.16	1	1	6	11	0.88	1	0	6	12	0.17
	Grade 2	0	0	4	2		0	1	2	3		0	0	1	5	
	Grade 3	0	2	9	6		0	2	7	8		0	0	10	7	
Pathologycal tumor stage	pT1	0	0	3	3	0.89	0	0	1	5	0.69	1	0	1	4	0.60
	pT2	0	1	6	6		1	1	4	7		0	0	3	10	
	pT3	0	0	4	4		0	0	4	4		0	0	3	5	
	pT4	0	1	8	6		0	3	6	6		0	0	9	6	
Tumor necrosis	Absent	0	0	9	8	0.23	1	0	6	10	0.38	1	0	4	12	0.22
	<50%	0	0	2	6		0	3	2	3		0	0	5	3	
	>50%	0	2	10	5		0	1	7	9		0	0	7	10	
Mitotic index	Low ≤5	0	0	4	4	0.84	0	1	3	4	0.31	0	0	4	4	0.52
	Intermediate 6-10	0	2	10	9		1	1	10	9		1	0	5	15	
	High >10	0	0	7	6		0	2	2	9		0	0	7	6	
Inflammation	Absent	0	0	8	11	0.32	1	2	7	9	0.91	1	0	6	12	0.46
	Present	0	2	13	8		0	2	8	13		0	0	10	13	
Fibrosis	Absent	0	1	11	13	0.48	1	2	8	14	0.69	1	0	8	16	0.51
	Present	0	1	10	6		0	2	7	8		0	0	8	9	
Resection margins	Negative	0	2	10	10	0.61	0	2	8	12	0.93	1	0	8	13	0.85
	Positive	0	0	11	9		1	2	7	10		0	0	8	12	
Recurrence	No	0	1	18	16	0.26	1	3	15	16	0.47	1	0	14	20	0.36
	Yes	0	1	2	4		0	1	1	5		0	0	2	5	
